# Wavelet Analysis Increases Sensitivity and Specificity of Spirography for Ambulatory Tremor Discrimination

**DOI:** 10.1155/2014/934746

**Published:** 2014-08-06

**Authors:** Veronika Kragelj, Dejan Georgiev, Zvezdan Pirtošek, Samo Ribarič

**Affiliations:** ^1^Institute of Pathophysiology, Faculty of Medicine, University of Ljubljana, Vrazov trg 2, SI-1000 Ljubljana, Slovenia; ^2^Department of Neurology, University Medical Centre, SI-1000 Ljubljana, Slovenia

## Abstract

The most frequently seen types of tremor are essential (ET) and parkinsonian tremor (PT) and in some patients clinical characteristics of these tremor types overlap. It is vital to distinguish between these two types of tremor in order to reach the right diagnosis and select the appropriate treatment. One of the widely used methods for tremor detection and discrimination, appropriate for a quick ambulatory assessment of the patient's tremor, is spirography. With spirography, the tremor can be observed through several parameters, for example, tremor spectrum and spiral image, which give useful information for its identification. Standard spirography parameters of ET and PT can overlap; therefore, these parameters are often not enough for identification of the observed tremor. To increase the specificity and sensitivity of spirography for PT, ET and normal, tremor free controls, we used the wavelet analysis with Morlet wavelet transform. To facilitate analysis, comparison, storage, and retrieval of spirography tremor records we also developed an integrated computer assisted spirography system that increases the convenience of outpatient tremor identification and follow-up. We conclude that wavelet analysis of spirography records increases the sensitivity and specificity of the method, thus, facilitating the distinction between ET and PT.

## 1. Introduction

Tremor is the most frequent movement disorder. There are several types of tremor, but the most common types are Parkinsonian (PT) and essential tremor (ET) [1]. In clinical practice, tremor is mostly diagnosed by using clinical examination only. However, by using only clinical examination, ET is accurately diagnosed in 50–63% of cases, whereas the PT in 76% of the pathologically confirmed cases [2]. The occasional overlap between different types of tremor makes diagnosing even more difficult. For example, more than 50% of patients with PT that have pathognomonic resting tremor also have postural tremor [2].

Patients with ET or PT have an increased activity in the cerebellothalamocortical circuit. In Parkinson's disease, the increased activity in the cerebellothalamocortical circuit is caused by the dopaminergic dysfunction of the pallidum triggers. In ET, the GABAergic dysfunction of the cerebellar dentate nucleus and brain stem, possibly caused by neurodegeneration in these regions, lead to tremulous activity within the cerebellothalamocortical circuit [3].

To reach the right diagnosis and select the appropriate medical treatment, it is necessary to discriminate between these two types of tremors. Additional methods for more precise tremor assessment have been used. For example: subjective scales for clinical assessment of tremor [4], functional tremor evaluation tests, tests for evaluation of tremor's impact on activities of daily living [5], physiological measurement methods—such as surface EMG [6], accelerometry [7], tremor tracking in electromagnetic field [8], video method [9] and also computer assisted spirography method (CAS) [10, 11].

Computer assisted spirography method (CAS) has already been used as an additional diagnostic tool for tremor discrimination for the last 20 years [10]. It is an appropriate method for a quick ambulatory assessment of the tremor, because it allows precise measurement of tremor amplitude and frequency, and also accurately quantifies the observed tremor in comparison with traditional clinical examination and other subjective methods. The tremor can be viewed through different parameters such as tremor spectrum, pressure spectrum, radius-angle transform, and velocity-time transform [10].

Although the standard parameters of CAS have been useful for intra- and interpatient tremor evaluation, the differentiation of tremors is still not perfect in some cases, especially because of overlapping different tremor types (e.g., ET and PT) and a variable expression of tremor amplitude over time [2].

At the Department of Neurology, University Medical Centre Ljubljana we included a new parameter for spirography analysis, the wavelet transform scalogram calculated with the wavelet analysis method using the Morlet wavelet transform in time-frequency representation, to increase the specificity and sensitivity of the CAS [12]. The Morlet wavelet transform is a type of continuous wavelet transform and the most popular complex wavelet used in practice, which mother wavelet is defined as

(1) $$\begin{array}{c} \psi \left(t\right)=\frac{1}{\sqrt[{4}]{\pi }}\left(e^{jw_{0}t}-e^{w_{0}^{2}/2}\right)e^{t^{2}/2}, \end{array}$$

where *w*_0_ is the central frequency of the mother wavelet.

To facilitate analysis, comparison, storage, and retrieval of spirography tremor records, we also developed an integrated computer assisted spirography system for patient record keeping that increases the convenience of ambulatory tremor identification, differentiation and follow-up.

## 2. Materials and Methods

### 2.1. Participants

Spirography records from fifty participants (20 with PT, 15 with ET, and 15 controls; 24 males and 26 females; average age was 61 years) were selected from the Department of Neurology, University Medical Centre Ljubljana database. The patients' and controls' hospital records were examined and selected by a senior neurologists [13, 14], so that patients with other parkinsonian syndromes (parkinson-plus syndromes, multiple-system atrophy, cortico-basal degeneration, iatrogenic parkinsonism), hepatic diseases, and alcoholism were excluded from this retrospective study. The study was approved by the Medical Ethical Committee of the University in Ljubljana and was in accordance with the Helsinki Declaration of 1975.

### 2.2. Patient Evaluation Protocol

**Figure 1:**
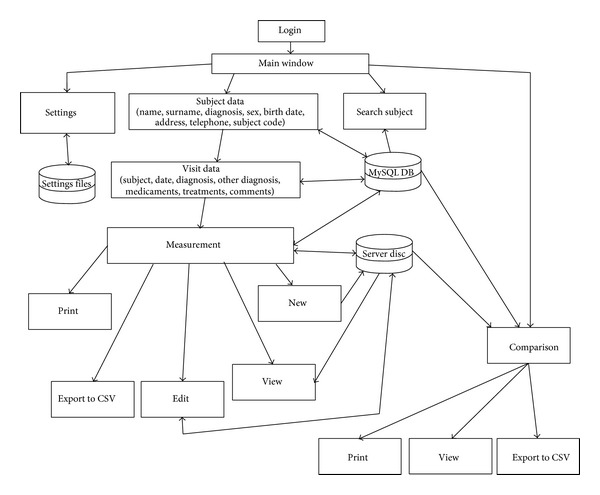
The structure presentation of the CAS application.

Previously, spirography data of patients with neurological diseases were stored on several computers using different recording, storage, data retrieval and analysis protocols. We developed an integrated computer assisted spirography system that facilitates analysis, comparison, storage and retrieval of patients' spirography tremor records (Figure 1) and has been routinely used at the Department of Neurology for several years. Hospital staff can access via the intranet the common patients' database using a single user interface for measurements, data analysis and retrieval of patient records. Thus, the system increases the convenience of ambulatory tremor identification, discrimination and patient follow-up. For proper administration of patient data, the user is first required to fill in subject and visit data input fields. Then the user can initiate a new measurement, view the recorded measurements, and edit or delete previously recorded measurements. The comparison between previously chosen measurements is another important part of CAS application. It allows comparing between two or more selected measurements through all observed parameters. All observed measurements can be exported to a comma separated value (CSV) file format. Tremor measurements can be made at a stationary computer, connected to the database via intranet, or on a laptop and later merged with the patient's database on the stationary unit.

The three spirography parameters, retrospectively evaluated in our study, were the drawn spiral image for qualitative evaluation [15], tremor spectrum [16], and a newly introduced parameter the wavelet transform scalogram [12].

During the measurement the participants sat on a chair and freehandedly drew a spiral on a programmed graphical tablet with the tablet's pen [17]. During the drawing process the participant was required to keep the pen in contact with the tablet's surface for the whole time. Patients with severe tremor were not always able to do that. However, the digital tablet can still track the pen's position even if it is raised for a few millimeters above the surface. This made the measurements on patients with severe tremor easier and also without the need for custom adjustments.

The spirography parameters (spiral images, tremor spectra, and wavelet transform scalograms) were evaluated visually by three experts, trained in spirography. Based on the results, the specificity and sensitivity of the three parameters for detection of PT, ET and healthy controls were calculated as follows [18]. For example, the sensitivity of spiral images for ET is the proportion of people with ET who will have a positive result (*a*/(*a* + *c*)) and its specificity is the proportion of people without ET who will have a negative result (*d*/(*b* + *d*)). Therefore, *a* is the number of patients with ET that test positive, *c* is the number of patients with ET that test negative, *b* is the number of persons without ET that test positive and *d* is the number of persons without ET that test negative.

## 3. Results

**Figure 2:**
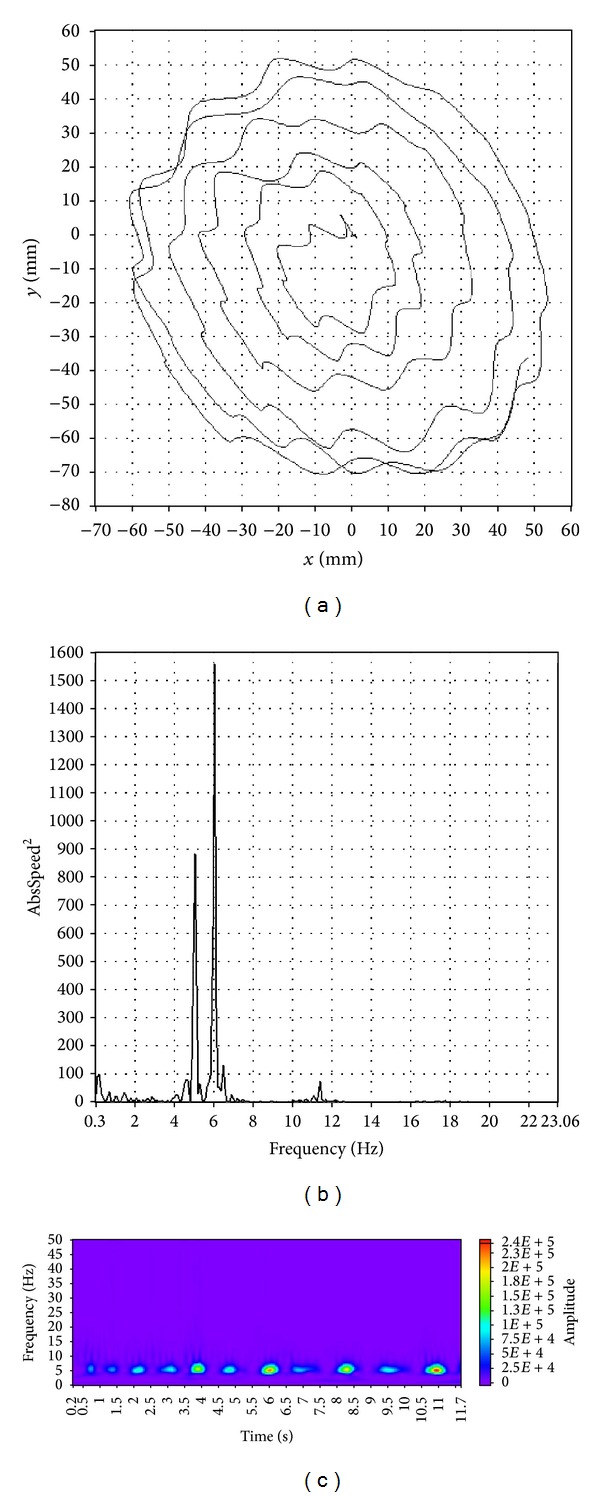
A typical example of spiral image (a), tremor spectrum (b), and wavelet transform scalogram (c) for an ET patient.

**Figure 3:**
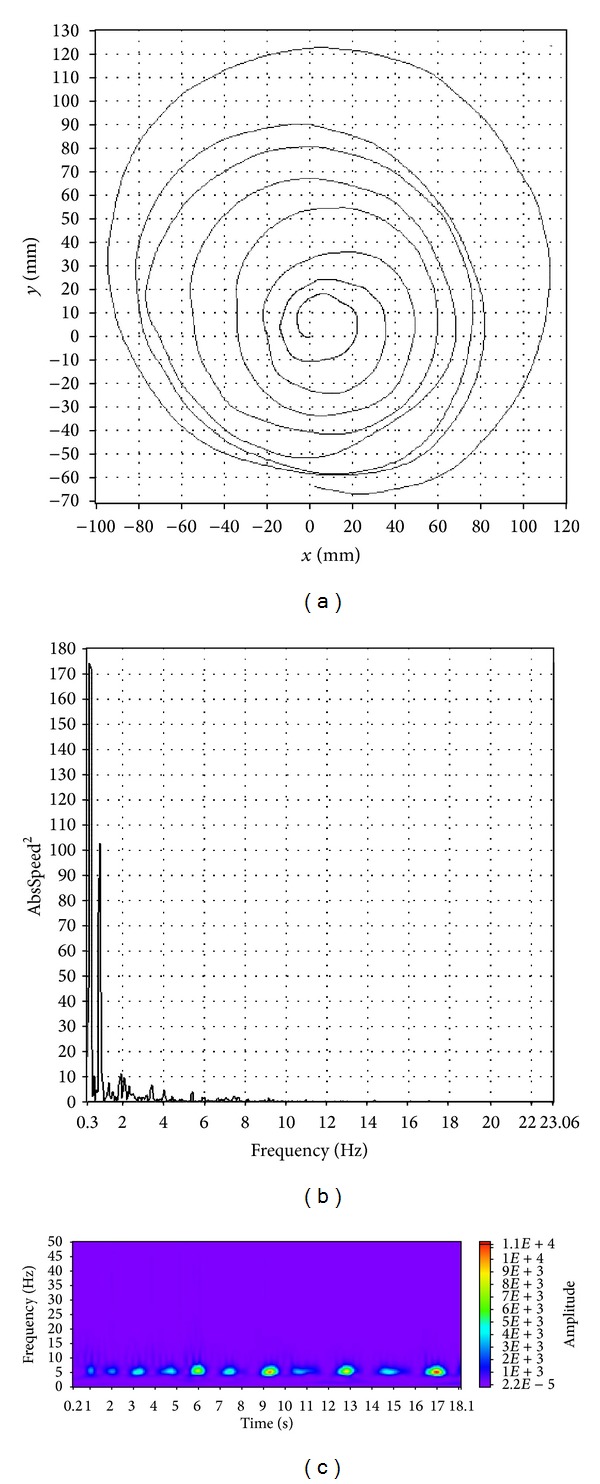
A typical example of spiral image (a), tremor spectrum (b), and wavelet transform scalogram (c) for a PT patient.

**Figure 4:**
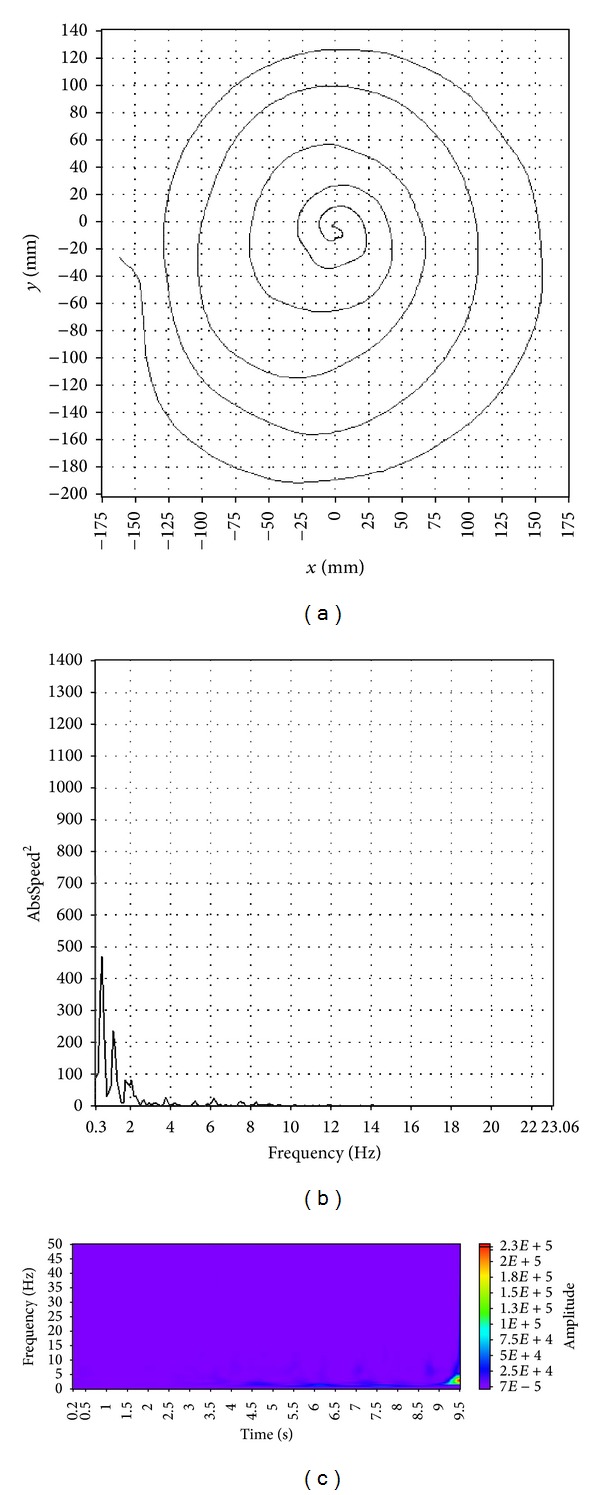
A typical example of spiral image (a), tremor spectrum (b), and wavelet transform scalogram (c) for a healthy, tremor free control.

Visual characteristics of spiral images, tremor spectra, and wavelet transform scalograms enable the distinction among patients with ET or PT and tremor free, healthy controls. A representative example of a spiral image, a tremor spectrum and a wavelet transform scalogram for an ET patient is shown in Figure 2. Spiral images, tremor spectra, and wavelet transform scalograms, for a PD patient and for a healthy control, are shown in Figures 3 and 4, respectively.

A patient with ET usually has a symmetrical and wavy spiral, a tremor spectrum with one or two dominant frequency peaks between 4 and 12 Hz and a wavelet transform scalogram filled with many oval shaped islands of activity (Figure 2). Patients with PD tremor (Figure 3) typically draw an asymmetrical spiral that is usually compressed at the base; their tremor spectrum has multiple peaks of activity up to 8 Hz and a wavelet transform scalogram with a continuously present activity during the time of drawing. Healthy, tremor free controls tend to draw a symmetrical spiral, have a tremor spectrum with peaks below 2 Hz and a wavelet transform scalogram with very little activity that is usually concentrated in the second half of the record (Figure 4).

We calculated the specificity and sensitivity of the three spirography parameters for diagnosing ET, PT and differentiating healthy controls. There was no difference among the patients' spirography parameters with regard to gender. The results are shown in Tables 1, 2 and 3 for ET, PT and controls, respectively.

### 3.1. Specificity

Diagnosing ET by visual examination of wavelet transform scalograms from spirography records had the highest specificity (0.99) and was similar to the specificity for differentiating controls by wavelet transform from ET and PT (0.97). Diagnosing PT only on the basis of the patient's frequency spectrum showed the lowest specificity (0.71). The lowest specificity in all three groups was noted for the tremor spectrum parameter (0.89, 0.71 and 0.80 for ET, PT, and controls resp.). Combining spiral image and tremor spectrum analysis improved specificity in all three groups compared to analysis with tremor spectrum or spiral image only (0.97, 0.82 and 0.86 for ET, PT and controls resp.) but did not reached the specificity for wavelet transform analysis only. Diagnosing tremor by combined visual analysis of all three parameters (i.e., spiral drawing, tremor spectrum, and wavelet transform scalogram) further increased the sensitivity and specificity of spirography for ET, PT, and controls. However, diagnosing by wavelet transform scalogram only was better than after combined analysis with all three parameters for ET and controls (0.99 versus 0.97 for ET and 0.97 versus 0.93 for controls). The combination of spiral image, tremor spectrum, and wavelet transform scalogram analysis slightly improved the specificity for PT diagnosis compared to wavelet transform scalogram only (0.92 versus 0.91).

### 3.2. Sensitivity

Combining spiral image, tremor spectrum, and wavelet analysis showed the highest sensitivity for diagnosing ET (0.91) compared to diagnosing PT or differentiating controls (0.88 and 0.87 resp.). The lowest sensitivity was for diagnosing PT by tremor spectrum only (0.53). Analysing spirography records by wavelet transform scalograms only showed the highest sensitivity for differentiating PT and controls (0.92 and 0.89 resp.). The highest sensitivity for ET was achieved with the combined visual analysis of spiral image; tremor spectrum and wavelet transform scalogram (0.91). However, combining spiral image, tremor spectrum, and wavelet transform scalogram analysis reduced the sensitivity for PT and for controls compared to diagnosing with the wavelet transform scalogram only (0.88 versus 0.92 for PT; 0.87 versus 0.89 for tremor free controls).

## 4. Discussion

The results of spirography tremor analysis show that, compared to spiral image and tremor spectrum evaluation, the wavelet transform scalogram has the highest sensitivity and specificity for detection of healthy controls and patients with ET and PT. Therefore, the wavelet transform scalogram is the most useful single spirography parameter to distinguish between these two common types of tremor. The main advantage of wavelet transform scalogram, compared to tremor spectrum, is that wavelets are localized in both time and frequency; it can detect amplitude change of a specific frequency with respect to time, whereas tremor spectrum is only localized in frequency and is useful for amplitude analysis of frequency component over the whole time span of a signal.

The sensitivity and specificity of all spirography parameters is lower for PT than for ET since spirography is more sensitive to action tremor (i.e., postural or kinetic tremors) than to resting tremors. PT is usually a resting tremor, most pronounced when the affected body part is completely supported against gravity. In ET, tremor of the hands is typically an action tremor. However, some patients with Parkinson's disease also have an associated action tremor [1]. Differentiation of PT from ET is also difficult because of an overlapping tremor frequency range (4–12 Hz for ET and 3–7 Hz for PT). The frequency of ET may decrease with age, thus, further complicating tremor diagnosis.

When the distinction between PT and ET with spirography is in doubt, a reliable diagnosis can be achieved by evaluating the wavelet transform scalogram or combining the visual analysis of tremor spectrum and spiral image. Evaluating tremor with all three spirography parameters simultaneously (i.e., spiral image, tremor spectrum, and wavelet transform scalogram) does not lead to a consistent, further increase in sensitivity, and specificity of spirography for PT, ET, and healthy controls.

**Figure 5:**
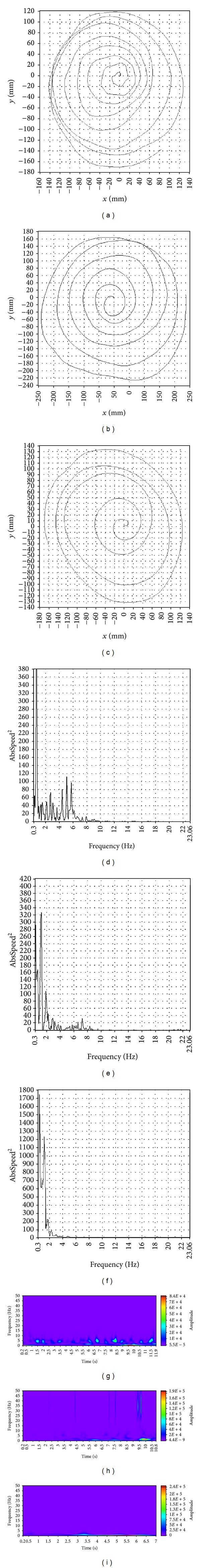
An example similar spirography parameters results for an ET ((a), (d), (g)), a PT ((b), (e), and (h)) and a healthy control ((c), (f), and (i)).

In Figure 5 we can see an example of similar spiral image and tremor spectrum parameters for PT and ET patients and a healthy, tremor free, and control participant. Therefore, the differentiation among PT and ET patients and the healthy control is possible only by the wavelet transform scalogram.

Previous spirography studies have evaluated tremor through different parameters [10, 19, 20], but none of them have used the wavelet transform scalogram nor compared the sensitivity and specificity of spiral image, tremor spectrum and wavelet transform scalogram for ET, PT, and tremor free controls. To our knowledge, this is the first spirography study that includes the wavelet transform scalogram as an additional parameter for tremor identification. It is also the first study that compares the sensitivity and specificity of spiral image, tremor spectrum, and wavelet transform scalogram in patients with ET or PT and in tremor free controls.

## 5. Conclusions

Because of the overlapping characteristics of different tremor types and variable expression of tremor amplitude over time, tremor differentiation is often difficult and conventional spirography parameters are often not enough for identification of the observed tremor. To improve tremor type differentiation with spirography, we introduced the wavelet transform scalogram. Compared to the spiral image and the tremor spectrum, the wavelet transform scalogram has the highest specificity and sensitivity for ET, PT, and tremor free controls. Thus, wavelet analysis increases sensitivity and specificity of spirography for ambulatory tremor discrimination.

## Figures and Tables

**Table 1:** Specificity and sensitivity for diagnosing ET (the number of patients is 15).

Spirography parameter(s)	Specificity ET	Sensitivity ET
Spiral image (S)	0.96	0.84
Wavelet transform scalogram (W)	0.99	0.87
Tremor spectrum (T)	0.89	0.73
S & T	0.97	0.82
S & T & W	0.97	0.91

**Table 2:** Specificity and sensitivity for diagnosing PT (the number of patients is 20).

Spirography parameter(s)	Specificity PT	Sensitivity PT
Spiral image (S)	0.75	0.65
Wavelet transform scalogram (W)	0.91	0.92
Tremor spectrum (T)	0.71	0.53
S & T	0.82	0.67
S & T & W	0.92	0.88

**Table 3:** Specificity and sensitivity for differentiating controls (the number of patients is 15).

Spirography parameter(s)	Specificity controls	Sensitivity controls
Spiral image (S)	0.79	0.59
Wavelet transform scalogram (W)	0.97	0.89
Tremor spectrum (T)	0.80	0.58
S & T	0.86	0.73
S & T & W	0.93	0.87
